# Influences and patterns of intimate partner violence among married Akha and Lahu women in northern Thailand

**DOI:** 10.1186/s12889-023-15162-4

**Published:** 2023-02-02

**Authors:** Onnalin Singkhorn, Tawatchai Apidechkul, Peeradone Srichan, Thanatchaporn Mulikaburt, Siwarak Kitchanapaibul, Anusorn Udplong, Panupong Upala, Chalitar Chomchoei, Fartima Yeemard, Ratipark Tamornpark, Pilasinee Wongnuch

**Affiliations:** 1 School of Nursing, Mae Fah Luang University, Chiang Rai, Thailand; 2 Center of Excellence for Hill Tribe Health Research, Mae Fah Luang University, Chiang Rai, Thailand; 3 School of Health Science, Mae Fah Luang University, 333 Moo 1, Ta Sud Subdistrict, Muang District, 57100 Chiang Rai, Thailand

**Keywords:** Intimate partner violence, Akha, Lahu, Hill tribe, Women, Source, Influence

## Abstract

**Background:**

Intimate partner violence (IPV) is a major global public health problem. Women are the principal victims of IPV, and some special populations have been particularly impacted. The Akha and Lahu women are vulnerable populations for IPV due to the modernization and changes of their culture and norms. This study aimed to understand premarriage factors related to IPV, including associated factors, influencers, patterns and impacts, in Akha and Lahu women in Thailand.

**Methods:**

A qualitative method was used to gather information among Akha and Lahu women who had experienced IPV in the previous year and were fluent in Thai. Women who had experienced IPV in the previous year and lived in the border area of Thailand and Myanmar were invited to provide information. A thematic analysis was used to extract information from the participants and develop findings.

**Results:**

A total of fifty-two married women were recruited into the study: 46.2% were Akha and 53.8% were Lahu. Those married Akha and Lahu women younger than 39 years found their partner through social media, had conditions before getting married, had high self-confidence, and tended to marry people from different tribes. While those aged 40 years and over met their husbands in village activities, were highly compliant with the norms of their culture, and married men from the same tribe. Three factors were detected as associated factors of IPV: cultural differences between partners from different tribes, substance use, and personality. Differences in age between partners, living in poor family economic status, and poor education were also detected as influencers of IPV. Four patterns of IPV were observed among Akha and Lahu married women: neglect, emotional abuse, verbal abuse, and physical abuse. Several patterns of the impacts were presented: children were neglected, especially in the preparation of daily food, having stress, having poor family relationships, and having children with unsuccessful lives in terms of education and getting a good job. Almost all married Akha and Lahu women had no particular expectations in their lives.

**Conclusion:**

Akha and Lahu women face IPV problems with several key influences and impacts. Effective implementations are required to monitor and reduce the problem in the Akha and Lahu families, especially where the women are younger than 40 years old and married to men from different tribes.

**Supplementary Information:**

The online version contains supplementary material available at 10.1186/s12889-023-15162-4.

## Introduction

Intimate partner violence (IPV) is a major public health problem and is currently identified as a major cause of human rights problems, especially in women [1]. IPV leads to health problems of individuals in a family and community [2]. It also greatly contributes to economic [3] and social problems [4]. IPV is clearly identified as a significant marker of having a critical point in life for children [5], women [6], and the elderly [7] in a family. The World Health Organization [WHO] reported that 35% of women worldwide had experienced violence from their partners in the form of physical violence, sexual violence, emotional abuse, and controlling behaviors [8]. Many factors have been identified as contributing factors of IPV, such as alcohol use [9], drug use [10], being young and of the same age [11], changes in gender roles [12], and poverty [12]. Under the crisis of the COVID-19 pandemic, IPV has increased its impact on both the frequency and level of violence [4]. Those who live in poverty [13], are minorities [14], and have a poor education [3, 15] are particularly vulnerable to IPV.

The hill tribe people in Thailand are a group of people who have their own culture, norms, and lifestyle [16]. In 2021, approximately 4.5 million hill tribe people lived in Thailand [17] within six main groups: Akha, Lahu, Hmong, Yao, Karen, and Lisu. All hill tribes living in Thailand have moved down from South China over several centuries [18], except the Karen, who originally lived in the border area of Thailand and Myanmar [19]. The Akha and Lahu are the largest and second-largest groups of hill tribe people, respectively, in Chiang Rai, Thailand [19]. Under globalization, many hill tribe people attend school and work outside their villages, including the Akha and Lahu people. Exposed to people outside their village, the Akha and Lahu people accept marriage with people from different tribes [20]. Mixing cultures, beliefs and lifestyles, the Akha and Lahu hill tribe people have started to engage in practices that are different from those of their tribes, such as drinking alcohol by females [15], and using Facebook or other social media to find partners [21]. While women are playing a key role in the family of maintaining the relationships of the family, especially through supporting children and the elderly.

Under the new living circumstances of the Akha and Lahu people including economic constraints and modernized lifestyles could cause IPV problems and become a new challenge for their families. The principal victims of IPV are women. Commonly, having IPV has not resulted in any positive impact for anyone [6, 11]. IPV could manifest its influences and patterns in different ways among women of different age groups. Currently, there is no scientific information regarding IPV in the Akha and Lahu families, especially in women. Therefore, the study aimed to understand the characteristics of the women before marrying, including associated factors, influences, patterns, and impacts of IPV in married Akha and Lahu women who lived in the border areas of Thailand and Myanmar.

## Methods

A qualitative method was used to elicit information from the participants. The study was conducted in fifteen hill tribe villages (eight Akha villages and seven Lahu villages) located in the border areas of Thailand and Myanmar in Chiang Rai Province, Thailand. Those Akha and Lahu married women who had experienced IPV in the previous year and were fluent in Thai met the inclusion criteria.

A question guide was developed from the literature review and discussion with people in the Akha and Lahu hill tribe villages. It was tested for validity and reliability before finalizing its use in the field. Three external experts (two medical anthropologists and one women health expert) were invited to review the question guide, which was aimed to validate the content of the questions. Moreover, four village headmen (two Akha village headmen and two Lahu village headmen) were invited to provide the comments especially in understanding of the questions. Afterwards, six married women (three Akha women and three Lahu women) who had experienced IPV were interviewed by the question guide.

Finally, there were seven questions in the final version: (1) Could you please give me your personal information?; (2) Could you please explain your relationship with your partner?; (3) Did you have any conflict or history of fighting with your husband during the previous year and what were the causes?; (4) Could you please share more details about the conflict or fighting, such as its influences and how long it lasts?; (5) Could you please give me the detail of the results or impacts of the conflict or fighting?; (6) Did you have any specific experience in dealing with the problem and what were the outcomes?; (7) What are the impacts of these problems on the people living around you? and (8) Do you have any expectations for your life?

After obtaining approval from the ethics committee for conducting research on human participants from the Chiang Rai Provincial Public Health Office, selected village headmen were contacted five days in advance. Essential information regarding the study was provided, including the inclusion and exclusion criteria for selecting participants in the study. On the date of data collection, all details of the study, including the objective and process of the data collection, were explained to the interested participants. Once the explanatory information step was completed, all participants were asked to provide informed consent on a voluntary basis.

An interview was conducted by three female researchers who were well-trained in qualitative research and had significant experience in conducting qualitative research (one psychiatric nurse, one medical anthropologist, and one behavioral health scientist). Moreover, all of the interviewers were familiar with the Akha and Lahu people based on their previous research experience.

Interviews were conducted in a private and confidential room located in the hill tribe village. A question guide was used as the major tool for running the interview. Before starting the interview, the interviewers introduced themselves and presented their personal information to the interviewees to make them familiar with each other. The question started from the basic information to the details of the conflict in their family. All interviews were recorded after special approval from the interviewee, including field notes. Each interview lasted approximately 60 min.

All records were typed and checked for errors before further analysis. The transcriptions were sent back to the research participants to check the accuracy of the information. Data were coded and the coding tree was developed by all researchers. All researchers were assigned to read and understand the meaning again. Meanwhile, the codes were transferred into the NVivo program (NVivo, qualitative data analysis software; QSR International Pty Ltd., version 11, 2015) to extract the themes. Thematic analysis was used to inform the findings. After developing the findings, all the researchers came together to again discuss the results to ensure that the forms properly presented the information of the study. Before making the final conclusion of the finding, all the results, including the extracted interview forms, were sent back to the interviewees again to confirm the findings. The findings were interpreted and are presented in the results section.

## Results

A total of fifty-two married women were recruited into the study: 46.2% were Akha and 53.8% were Lahu; 41.7% of the Akha women and 42.9% of the Lahu women were married to men of different tribes. More than half were aged 40 years and over, 65.4% had never attended school, 44.3% were daily-wage earners and 57.9% had a monthly income ≤ 4,999.

The findings were presented in two important stages: characteristics before marriage, and characteristics of IPV after getting married and its impact. Five important characteristics before marriage were detected: approaches to find a partner before marriage, conditions before marriage, self-confidence, compliance with norms and culture, and marrying with different tribes. Four characteristics of IPV after getting married were formed and presented: associated factors of IPV, influence of IPV, pattern of IPV, and impact of IPV (Table 1).

While, three specific traits were detected as factors associated with IPV: difference of culture between partners, substance use, and personality. Three sociodemographic characteristics were detected as the influence of IPV: difference in age between wife and husband, poor family economic situation, and low education (Table 1).

**Table 1:** Structure of the study fining presentation

Characteristics	Factors	Sub-factors
(I) Characteristics and conditions before marriage	(i) Approaches to find a partner before marriage,	
	(ii) Conditions before marriage,	
	(iii) Self-confidence,	
	(iv) Compliance with norms and culture,	
	(v) Marrying with different tribes.	
(II) Characteristics of IPV after getting married	(i) Associated factors of IPV	(a) Difference of culture between partners.
		(b) Substance use
		(c) Personality
	(ii) Influence of IPV	(a)Difference in age between wife and husband
		(b)Poor family economic situation
		(c) Low education
	(iii) Pattern of IPV	
	(iv) Impact of IPV	

### I. Characteristics before marriage

Of the women who suffered IPV, there were many characteristics and personality differences between those younger than age 39 and those aged 40 years and older, such as the approach to finding partners before marriage, having conditions before getting married, self-confidence, compliance with norms and cultures, and marrying into different tribes.

#### (i) Approaches to finding a partner before marriage

The married Akha and Lahu women aged 39 years and younger and 40 years and over had different approaches for finding their partners. Those aged 39 years and younger use various social media platforms for finding partners, while those aged 40 years and above use traditional methods such as meeting at new year party, through a matchmaker, etc.

A 32-year-old Akha married woman said [P#38].

“I used a chat-dating application many years ago. I chatted with my boyfriend while he worked in Taiwan for two years before I moved to work in Taiwan. He is my husband today.

A 56-year-old Lahu woman said [P#32].

“I was born at Myanmar. I was a single child in a very poor family. I met my husband while we were at the new village new year ceremony.

#### (ii) Conditions before marriage

Those married Akha and Lahu women aged 39 years and younger placed many conditions on their husbands before marriage, while those aged 40 years and over did not place any conditions on their husbands.

A 24-year-old Lahu woman said [P#48].

I had discussed with my husband in the earlier days of our marriage that he will not assault me. If he does so, I will not live with him because we both have a job.

A 32-year-old Akha woman said [P#38]

I have requested to my husband three issues before we married. First, do not assault me. Second, all assets will be shared equally in case we have to separate. Three, no mistress

#### (iii) Self-confidence

Akha and Lahu married women younger than 40 years old had more self-confidence compared to those older than age 40 in terms of making decisions about their partner lives. The older women had no thoughts on presenting self-confidence to their husbands.

A 18-year-old Akha woman said [P#4]

I have had many partners, I have never been assaulted. I had been assaulted from my husband a few months ago. It was my first time, I tell myself that I would not be patient. I have left him, I do not like him anymore.

A 26-year-old Akha woman said [P#39].

“In my idea, we all have same equity. I would not accept the mistake from my husband. If he did wrong he has to be responsible for what he did. I have no idea about acceptance of anything of husband’s mistake. We always talk based on a good and acceptable reason while we have a conflict.”

#### (iv) Compliance with norms and cultures

Those Akha and Lahu married women aged 40 years and over followed the norms and their cultures more strictly than those aged 39 years and below.

A 61-year-old Akha woman said [P#7]

I have been married with my husband more than 30 years. We have had many conflicts throughout our partner-life, but I have no better option. I have to live with him. In Akha culture, a woman cannot answer to her husband in any way. A woman has to live with her husband until she dies.

A 58-year-old Lahu women added [P#11]

In Lahu culture, a man can have more than one wife or polygamy. Then, if we get married to any man, we have to live with him without condition.

A 18-year-old Akha woman said [P#4]

I did not follow our original culture on having partner. I and many of my friends have boyfriends when we attend school. I personally do not care if I need to divorce my husband, but you know it is not possible among the women in previous generations. I do not agree with this.

A s [P#52]

All my friends, we have boyfriends while in school, and nobody has a problem with our parents. We thought that we all have the same rights in having boyfriends or girlfriends. Moreover, my mom said that we can do what we are doing now because we changed our religion from traditional religion to Christian. If we still have our tradition religion, we have to follow many things and many steps for having husband.

#### (v) Marrying with different tribes

Many Akha and Lahu married women aged 39 years and younger reported that marrying with different tribes was common, while of those aged 40 years and above, there were no reports of having been married with someone from another tribe.

A 25-year-old Lahu woman said [P#9]

I am Lahu, but my husband is Thai. I do not mind. We married while we were working together in Bangkok. However, we have lots of problem between us.

A 27-year-old Akha woman said [P#3]

My husband is Lahu. We met five years ago while attending a school. Basically, our parents supported us in getting married. Sometime we have conflict, and I am not too happy with my life now.

### II. Facing IPV after getting married

Facing IPV among the Akha and Lahu women after getting married were presented in different stages: associated factors of IPV, influence of IPV, pattern of IPV, and impact of IPV.

#### (i) Associated factors of IPV

Three associated factors of IPV were detected among Akha and Lahu married women: differences in culture between partners, substance use, and personality.

##### (a) Difference of culture between partners

IPV was found in many Akha and Lahu women who married men from different tribes experienced IPV more than those who married within the same tribe.

A 25-year-old Lahu woman said [P#9]

I feel that I am undervalued compared my husband who is from a different tribe. I have got many troubles with my mother in law. Almost every morning, I and my mother in law have at least something from different directions. The result is I and my husband have many things to discuss and I feel unhappy.

A 27-year-old Akha woman said [P#3]

I am Akha but my husband is Lahu. From the very beginning after getting married, we were very happy. Years later, I felt that we have many things different. Today, we have many different lifestyles including perceiving in my husband tribe relevant practices.

##### (b) Substance use

Substance use was detected as a significant source of IPV among Akha and Lahu married women. Using a substance by either spouse or both wife and husband was a poisonous source of IPV in the family.

A 31-year-old Lahu woman said [P#23]

My husband told me that he used methamphetamines for three years, and whenever he did not take a drug, he always asked trouble questions. We have frequent conflicts since 2019 during the COVID-19 pandemic. During the pandemic, we could not get income, then my husband could not afford to buy drugs, and there was conflict between us. I truly do not like it.

A 22-year-old Lahu woman said [P#24]

I sometime use methamphetamines. Meanwhile, my husband takes amphetamines and drinks alcohol on some days. I felt that we have many troubles in my family.

##### (c) Personality

The personality of the wife and husband were also detected as one of the associated factors of IPV experienced by Akha and Lahu married women. An aggressive or irritable personality of the husband was found to be a source of IPV against his wife, while irritability in women was also found to be a leading personality trait associated with IPV in Akha and Lahu families.

A 50-year-old Akha woman said [P#27].

“I have a very aggressive and irritable man. I can’t even ask him where he was in the day time? Asking the question because I worry whether he use drugs with his friends in the village. However, do you know what he responds to me. He told me to stop taking with him if I do want to get kicked by him. It’s very bad right!”

A 40-year-old Lahu woman said [P#30]

Yes, I am slightly talkative, and I just want to ask my husband many questions. He said, I was a woman who could not talk properly. He said that I can talk every bad thing in the front of our kids. With this, we have had many times conflicts. He also asked me not to look through his pocket. Sometime, he says bad words to my parents as well.

#### (ii) Influence of IPV

Three influencers of IPV among the Akha and Lahu married women were detected: difference in age of partners, being from a poor family, and level of education.

##### (a) Difference in age between wife and husband

Age differences between wives and husbands could be an influencing factor of IPV. If either the husband or wife was older than the other, this was found to lead to IPV in married Akha and Lahu women.

A 38-year-old Akha woman said [P#44]

I am now 38 years, but my husband is 50 years. I feel that we have many things different particularly how to care for our kids. I need to send them away to study in city but my husband disagrees. We also have differences about our future life. I want to start trading, but my husband likes to work on a farm. We have started discussing serious points in previous weeks. I think we both are not feeling happy now with our current life.

A 40-year-old Lahu woman said [P#45]

My husband is older than me by 12 years. When I married with him, I did not have agreement from my parents, but I loved him. Today, I realized that different ages make many troubles of life. I want to get good food in the city, but my husband did not. I want some time to see a movie, but he did not. We have got many times in arguments, and I am thinking the problem will greater and greater.

##### (b) Poor family economic situation

A 65-year-old Lahu woman said [P#28]

I grew up with a very poor family. My father and mother used opium. We had no rice to eat, we had to eat corn for our daily life. I have not had a good life in my whole life. Even after marrying with my husband, we still live as poor people. He drinks alcohol a lot and started fighting with me. So bad right!

A 63-year-old Akha woman said [P#35]

I married my husband without love. My mom told me just to get married then everything will be going well. I do not think so. With very poor status and living with the one who we do not love, I truly suffer. I and my husband have arguments and fights almost every week. Definitely, I have got hurt and pain.

##### (c) Low education

Education was detected as another influencer of IPV among Akha and Lahu married women; those with a poor level of education experienced more IPV.

A 41-year-old Akha woman said [P#16]

I married with my husband who is Yao. I graduated primary school while my husband graduated university. My mother in law does not like me because I have graduated just primary school. I have many times conflicts with here and my husband. My husband, he loves his mom and looks down on me sometimes.

#### (iii) Pattern of IPV

Four forms of IPV occurred among Akha and Lahu married women: neglect, emotional abuse, verbal abuse, and physical abuse. Some married women have been neglected from care and financial support from their husbands and mothers-in-law. Neglect by the husband while sick, particularly after fighting, was commonly reported by Akha and Lahu married women. A consequence of the conflict is they are not supported financially by their husbands.

Emotional abuse was another major pattern of IPV that experienced by Akha and Lahu married women. Emotional abuse always occurred with verbal abuse. Married Akha and Lahu women reported that while having arguments with their husband, they felt unhappy and had many thoughts about what the husband said and become very worried on some matters. The worry from the emotional and verbal abuse made it difficult to sleep. Some cases reported that after getting into heavy arguments with their husbands, they moved back to their parents’ home, which caused their parents to feel unhappy. Emotional abuse and verbal abuse also impacted their children. Loud, heavy arguments always make children fearful and uncomfortable, including feeling unsafe in the situation. Emotional abuse and verbal abuse very often occurred while the husbands drank alcohol.

Finally, physical abuse took place on three different levels: mild, moderate, and severe. Most of the physical abuse occurred when one spouse or both wife and husband used drugs or alcohol. The mild form of physical abuse occurred when someone did not want to fight with the other and could easily stop the incident. If it lasted a long time, moderate and severe physical abuse was reported. The most moderate and severe forms of IPV were reported among Akha and Lahu married women aged 25–35 years. Moderate and severe forms were also reported among women whose husbands were from different tribes and who had some personal traits, such as being talkative. The most severe cases (broken arm and head) were reported by those who used amphetamines and alcohol, and who lived in poor families.

#### (iv) Impact of IPV

All IPV experienced by married Akha and Lahu women was reported to have different impacts on the people living around them. Children were neglected from being taken care of, especially in preparing daily food. Children also experienced stress from their parents’ fighting and feeling insecure. Poor family relationships among family members were detected as another impact of IPV. Poor family relations among Akha and Lahu married women led to the unsuccessful lives of their children in terms of education and not getting a good job. Almost all married Akha and Lahu women reported that they did not have any special expectations of their lives. Some reported that even though they did not have happiness in their lives, they had to move on with their lives for their children. A few cases reported that they needed to get medicine after fighting with their husbands due to sleeping and stress problems.

A 56-year-old Lahu woman said [P#32]

Two of my sons told me that they suffered while our family had a conflict. Both of them said that while they saw I and my husband had argued, it made them insecure and very sad.

A 61-year-old Akha woman said [P#7]

I always need to get medicine after having a conflict with my husband. I do know that it is not good, but if I do not take medicine I would not sleep.

## Discussion

Akha and Lahu women had some characteristics or styles before getting married, such as how they found their partner, imposing conditions before marriage, being self-confident in decision-making, especially with their partner, compliance with norms and culture, and deciding to marry a man who was from a different tribe. Different cultures between partners, substance use, and some personality traits were detected as sources of IPV. The influencing factors of IPV included age difference between wife and husband, poor family economic circumstances, and a low level of education. The pattern of IPV was found to range from mild to severe: neglect, emotional abuse, verbal abuse, and physical abuse. The IPV experienced by the Akha and Lahu married women affected the women the other people who were living in the family, especially children. Among the Akha and Lau married women who experienced IVP, they did not have clear, positive expectations of their lives.

There were several personal characteristics of Akha and Lahu married women that influenced the occurrence of IPV after marrying. Married women aged 39 years and younger and those aged 40 years and over presented some key differences. Those aged 39 years and younger met their partners through social media and had high confidence in choosing their partners with some conditions established before marrying. Those in the older age group met their partners through a traditional ceremony in their village, which took place occasionally, and there were few options for who to marry. Those who were older complied with their traditional norms and cultures more than those of younger ages. Moreover, those younger tended to have married men who were from different tribes. The change in the approach to find their partners and other characteristics between the two generations of Akha and Lahu married women could be an effect of socioeconomic development and globalization. The flow of current mainstream social media has a great impact on finding partners of Akha and Lahu women and is a major route toward IPV later. Although starting a new family begins with a woman and a man, a family does not consist of only two persons but is still a part of people belonging to their original families and cultures.

Having different cultures between Akha and Lahu partners, substance use, and some personality traits were found to be the main sources of IPV in Akha and Lahu married women. This coincides with a study conducted in the Latino population [22], which found that some cultural characteristics were detected as protections against IPV. A study in Canada reported that aboriginal women who intended to practice their traditional culture and norms were at a greater risk of IPV than nonaboriginal women [23]. Many countries in Africa were reported as having high rates of IPV according to the norm of male domination within their specific cultures [24]. A meta-ethnographic study reported that substance use was closely related to IPV at different contextual levels under the dynamics of power and control in different populations [25]. Golinelli et al. [26] reported that IPV had a greater impact when both women and partners used substances. Moreover, those of some races faced a larger impact from IPV when the husbands used substances [27]. A study in Thailand reported that alcohol use was associated with IPV [28], while a study in the Lahu people also reported that alcohol use was a risk factor for IPV [3, 29]. Clemnents et al. [30] reported that some traits, such as the aggressiveness of men, were associated with IPV against women. A study in the United States confirmed that some personality traits, including partner relationships, were associated with IPV [31]. Therefore, Akha and Lahu married women were potentially impacted by IPV due to the role of norms, culture, and substance use, as well as some personality traits of their partner.

The difference in age between partners, poor economic level and low education status were detected as influencers of IPV among Akha and Lahu married women. A study in 30 developing countries reported that most IPV occurred 3.5 years after getting married and that the gap in age between partners was a contributor to IPV [32]. A study in a developing country reported that women who had regular income were at low risk of IPV compared to those who did not [33]. A systematic review reported that poor economic conditions, especially among ethnic populations, were a major contributing factor of IPV [34]. Regarding education, a study in India reported that women who had less education than their husbands had a significantly greater risk of suffering IPV than those who had the same or higher education than their husbands [35]. Another study conducted in India and Bangladesh reported that women who had a low or the same level of education as their husbands faced a risk of IPV more than those who had a higher level of education than their husbands [36]. A study in Malavi confirmed that women with less education had a greater risk of experiencing IPV [37]. The information obtained from several studies [38–40] showed that most of the Akha and Lahu people had low socioeconomic status (SES), and young married women could be greatly impacted by IPV if their SES acted as the influencer.

There were different patterns and impacts of IPV presented among Akha and Lahu married women in Thailand. Neglect, emotional abuse, verbal abuse, and physical abuse were the key patterns of IPV, while the impacts included neglecting children in the family, having poor family relationships, having no happiness in life or any specific hope, and needing medical care in some cases. A study conducted in India [41] reported that the major form of the IVP was physical violence. A study in Thailand confirmed that emotional abuse, verbal abuse, and physical abuse were commonly reported by women suffering IPV [42]. Another study in Thailand reported that children were major victims of IPV [43]. The pattern of IPV in this study was similar to that reported by Ann et al. [44], who reported the pattern of IPV according to the dimension and severity of IPV. Among pregnant women in Nigeria [45] who did not report their suffering from IPV, but those who voluntarily reported their experience found that there were similar patterns that occurred among Akha and Lahu women in Thailand.

A few limitations were found in the study. First, during the interviews, some participants had difficulty expressing their feelings and suffering, which is a limitation of language. Therefore, clear communication was required by asking questions in different forms. Second, interviews in the village hall maintained privacy, but some women were uncomfortable expressing their pure feelings and suffering. Helping them relax during the interview and obtaining real information were very important. Last, with the specific characteristic of a qualitative study in collecting data in two hill tribes, it could not be generalized to the whole hill tribe population in Thailand.

## Conclusion

The Akha and Lahu women 39 years of age and younger and those 40 years old and above differ in how they find their partners, their self-confidence and compliance with their norms and culture. Akha and Lahu married women are encountering IPV problems, particularly those who have married men who belong to different tribes, who engage in substance use, and who have some personality traits. Moreover, age differences between partners, having a poor family economic status, and a low level of education were identified as major influencers of IPV among Akha and Lahu married women. Akha and Lahu married women with IPV are neglected, emotionally abused, verbally abused, and physically abused by their husbands. The impacts of IPV are not limited to women but also their children in the form of unsuccessful lives later on. Because of the IPV, married Akha and Lahu women do not have positive expectations about their future lives.

Further study of the impacts of IPV on children, disabled persons, and elderly individuals in the family needs to be investigated. Intensive or clinical research should be considered to operate for seeking the solutions of the IPV problem in these communities especially in developing a community-based care and health seeking channel. The findings could be used for forming the policy or guiding public health practices. Moreover, effective implementation of strategies to reduce the incidence of IPV among Akha and Lahu women is also urgently required by integrating work plans from stakeholders, especially from those who are experts in the field.

## Electronic supplementary material

Below is the link to the electronic supplementary material.

Supplementary Material 1

## Data Availability

The datasets used and/or analyzed during the current study are available from the corresponding author on reasonable request.

## Abbreviations

COVID-19: Coronavirus disease 2019
IPV: Intimate partner violence
