# Supplementary material for: Influences and patterns of intimate partner violence among married Akha and Lahu women in northern Thailand
Source: BMC Public Health. 2023 Feb 2;23:228. doi: 10.1186/s12889-023-15162-4 (PMC9893967; doi:10.1186/s12889-023-15162-4)
Supplement: Supplementary file 1 — Supplementary Material 1 [file 12889_2023_15162_MOESM1_ESM.docx]

**Question guide**

1) Could you please give me your personal information?

2) Could you please explain your relationship with your partner?

3) Did you have any conflict of fighting with your husband previous year and what were the causes?

4) Could you please give me more detail on your conflict or fighting such as its influencer, lasting?

5) Could you please give me the detail of the results or impacts from the conflict or fighting? 6) Did you have any specific practice to dealing with the problem? and what were the outcomes?

7) Any impacts to people living around you from your problem? 8)Do you have any expectations in your life?
